# Influence of different preservation methods on the mechanical in vitro stability of bones: a comparative study using porcine metatarsals

**DOI:** 10.1007/s00402-026-06226-2

**Published:** 2026-03-13

**Authors:** Martin Bäumlein, Moritz Kühlein-Lehr, Alexander M. König, Volker Ziller, Steffen Ruchholtz, Jürgen R. J. Paletta, Vanessa dos Santos

**Affiliations:** 1 Marburg University, Marburg, Germany; 2 Center for Orthopaedics and Trauma Surgery, University Hospital Gießen and Marburg, Marburg, Germany; 3 Department of Diagnostic and Interventional Radiology, University Hospital Gießen and Marburg, Marburg, Germany; 4 Clinic for Gynecology and Obstetrics, Department of Endocrinology, Reproductive Medicine and Osteology, University Hospital Gießen and Marburg, Marburg, Germany

**Keywords:** Three-point-bending-test, Fresh frozen, Formalin, Thiel, Ethanol–glycerol, Biomechanics

## Abstract

**Introduction:**

Large quantities of bone specimens are required for the mechanical testing of orthopedic and trauma implants. Currently, fresh-frozen or chemically preserved bones—such as formalin-fixed—are commonly used. This study aimed to evaluate the effects of different conservation methods on mechanical bone stability under standardized conditions.

**Materials and methods:**

Porcine metatarsals were divided into seven groups based on the preservation technique: fresh, fresh-frozen (− 18 °C), frozen with repeated freeze-thaw cycles, vacuum-sealed and refrigerated (4 °C), and chemically fixed using formalin, Thiel-fixation, or glycerol-based methods. All specimens underwent three-point bending tests to assess mechanical stability. Following induced fractures, bones were stabilized with a plate osteosynthesis (DCP^®^ small fragment set, Synthes^®^, Germany) and retested. Bone mineral density (BMD) was also measured via dual-energy X-ray absorptiometry (DXA).

**Results:**

Results revealed no significant differences in BMD, maximum load, or breaking strength between groups. However, chemically fixed bones—especially formalin and glycerol—showed significantly reduced deformation, indicating increased stiffness. Additionally, chemically fixed specimens exhibited more consistent fracture patterns compared to fresh or physically preserved bones. After osteosynthesis, bones preserved with glycerol showed a significant increase in both maximum load and breaking strength.

**Conclusions:**

While physical preservation methods had no notable effect on mechanical stability, chemically fixed bones exhibited altered deformation characteristics and should therefore be used selectively, depending on the research question.

## Introduction

Due to advances in medicine and the development of new treatments and implants in the field of trauma surgery, the demand for bones for mechanical testing and implant development has increased. A search in the literature using the keywords “mechanical testing” and “bone” yields approximately 18,000 results on PubMed^®^ and 196,000 on PubMed Central^®^. This indicates that a large number of bones are required not only for research but also for development and teaching, raising the question of what sources of bone are available for mechanical testing and in sufficient quantities.

Generally, there are several options including fresh frozen human bone, embalmed human bone or artificial bone. Reference here should be the fresh human bone. However, this is problematic, as the mechanical properties begin to change immediately after removal. Studies report a 10% decrease in stiffness within the first 24 h post mortem, which can be avoided through freezing [1, 2].

A temperature of − 20 °C is considered suitable to preserve the mechanical properties of human cadaveric bones [3, 4]. Regarding long-term storage, earlier studies [5] found no significant mechanical deterioration after up to 5 years of cryopreservation at − 80 °C. Freezing also proves to be robust with respect to repeated freeze-thaw cycles, at least in terms of stiffness [2, 6].

However, fresh-frozen bones are difficult to obtain in large quantities, are more expensive and require more complex handling. Therefore, chemically fixed specimens (primarily formalin-fixed bones) are frequently used in biomechanical testing. Yet their suitability for such tests remains controversial. It is well known that fixatives can alter collagen structures in soft tissues, thereby affecting mechanical properties [7–9].

Formaldehyde, for example, blocks amino groups in peptides and creates hydroxymethylene cross-links [10, 11]. Additionally, formic acid might be formed, which alters the bone density by dissolving calcium components and therefore weakens the bone.

Burkhart et al. [12] reported increased stiffness in formalin-fixed bones, although this was not reflected in bone mineral content. Wilke et al. [13] found that formalin-fixed spine specimens did not replicate in vivo conditions—the range of motion decreased by approximately 80%, and the neutral zone by up to 96%. These alterations in joint motion are likely due to fixation-induced changes in the mechanical properties of the ligaments and intervertebral discs. On the other hand, Topp et al. [14] found no differences in deformation stiffness or screw pull-out strength between fresh-frozen and formalin-fixed femora. However, both were significantly weaker compared to composite bones, which are designed to represent the quality of young, healthy human bone. Elfar et al. [15] described near-physiological values for torsional, axial deformation, and lateral bending stiffness, as well as cancellous screw pull-out strength. Nonetheless, most of these studies only compare two preservation methods and use different mechanical test protocols, making it difficult to evaluate individual methods systematically.

While there is general agreement that freshly frozen bones are most suitable for mechanical testing due to their similarity to fresh specimens, only a few studies address the use of formalin-fixed or synthetic bones. Even fewer consider other chemical preservation techniques, such as Thiel or glycerol.

This study presents a comparative evaluation of various preservation methods and analyses their effect on mechanical properties under standardized conditions.

## Methods

### Specimens

Metatarsal bones of domestic pigs, which have reached skeletal maturity, were used in this study, due to their uniform size, shape and mechanical stability [16]. The bones were prepared from fresh slaughtered pigs feet, obtained from a local slaughter house, which did not require animal ethics approval. Each preservation group consisted of 12 specimens (*N* = 12), resulting in a total of 84 bones included in the study.

### Preservation methods

Metatarsal bones III and IV were dissected from soft tissue; their dimensions were measured and randomized into 7 groups as followed: (1) fresh bones (control), (2) storage at − 18 °C, (3) storage at − 18 °C with 7 freeze/thaw cycles (a cycle consisting of 8 h thawing and 24 h re-freezing), (4) stored at 4 °C in vacuum, (5) fixed with formalin according to the local anatomic institute (1000 ml water, 4330 ml Ethanol, 433 ml 37% Formalin solution and 233 ml Phenoxitol), (6) Thiel-fixation [17] and (7) ethanol–glycerol fixation [7]. Bones treated like this were incubated over a period of 6 months, except the vacuum group, which was stored for 2 months in their anaerobic surroundings.

### Bone density

Every specimen underwent bone mineral density (BMD) measurement via dual-energy X-ray absorptiometry (DXA) [HOLOGIC^®^ Horizon W (S/N 301261 M)] according to the manufacturers instructions—before and after the respective preservation technique. The specimens were positioned standardized on the plantar surface and the scanning direction was from distal-to-proximal. There were three standardized measuring points (distal metaphyseal, diaphyseal and proximal metaphyseal) for the scan.

### Fracture model & biomechanical testing

**Fig. 1:**
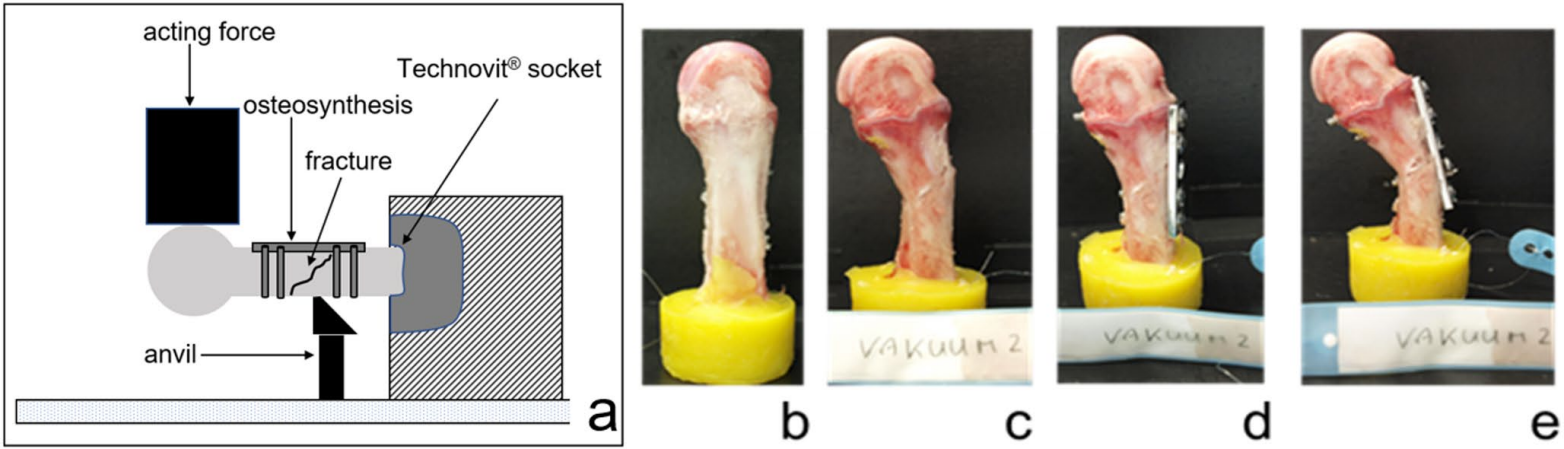
Schematic setup and example of a biomechanical test series in the three-point bending test. **a** Experimental setup with the metatarsal bone embedded in a Technovit^®^ socket. The setup was designed so that the anvil served as a support positioned between the second proximal screw and the fracture gap, while the load was applied distal to the gap; a milled groove prevented direct loading of the plate. **b** Specimen mounted in the testing machine, **c** generated standardized oblique fracture, **d** fixation with a DCP^®^ small fragment plate, **e** repeated fracture testing after osteosynthesis

Bones (native and after osteosynthesis) were mechanically tested using a modified 3-point bending method as described [18, 19] (Fig. 1). Briefly: Bones were embedded in a fixation device (14 mm of the proximal bone) using Technovit^®^ 3040 (Heraeus, Wehrheim, Germany) according to the manufacturers instruction. All specimens were allowed to equilibrate to room temperature and were kept moist with saline irrigation during preparation, surgical procedure and biomechanical testing.

Load to failure was applied with a constant speed of 100 mm/min using an Instron^®^ 5566 material testing system (Instron^®^, Canton, MA, USA). Only shaft fractures similar to AO A2.2 were used in the study and evaluation.

### Implants & fracture treatment

Fractures created as described above, were dorsally reduced and fixed using a mini-plate (DCP^®^ small fragment set, thickness 2.0 mm, 5 holes) and 2.3 mm screws (Synthes^®^ Umkirch Deutschland) (Fig. 1). This simple, non-locking, conventional plate system was selected to minimize implant-related variables and to allow the mechanical influence of the preservation method to be isolated. Although the implant is no longer in clinical routine use, it was available in sufficient quantity and enabled standardized fixation across all specimens. As the objective of this study was to assess the mechanical behavior of the bone itself, rather than implant performance, the choice of implant did not limit the relevance of the results. All fixations were carried out standardized by one surgeon. The plate was positioned dorsally on the bone in order for the middle hole to come to place right over the fracture. Two non-locking screws were placed bicortically on each side of the fracture, as commonly used [20], right-angled to the plate.

### Data acquisition and statistical analysis

Prior to the experiment sample size estimation was performed using G*Power 3.1. The calculation was based on the primary outcome of maximum load in a comparable 3-point-bending-test previously reported [18, 19]. A suitable effect size of f = 0.45 at alpha-level of 0.05 and a statistical power of 0.8 for a one-way ANOVA with seven independent groups indicated that 12 specimens per group were required.

Data were collected using Bluehill^®^ II software, series 5500 (Instron^®^, Canton, MA, USA). A force-distance-diagram (N/mm) was recorded.

Stiffness was calculated at the maximum load due to the nonlinear nature of the force-deformation curve. In bone testing, the material typically enters the plastic deformation phase under higher loads, which alters the stiffness. Calculating stiffness at maximum load provides a more accurate representation of the bone’s mechanical stability and structural integrity under realistic conditions. This method has been commonly used in biomechanical studies to better capture the nonlinear material response and ensure a more precise evaluation of the bone’s mechanical properties.

Mean value and SD were calculated for intact bones and for each test group using SPSS 15.0 (IBM^®^ Deutschland GmbH; Ehningen, Germany).

P-values represent results of overall group comparison using Kruskal–Wallis or ANOVA, depending on the results of the Kolmogorow–Smirnow–Test. Post hoc comparisons were adjusted for multiple testing using the Bonferroni correction—analyzing and comparing each preservation group with the fresh bones as control group. Statistical significance was set at a value of *p* < 0.05 and are labeled in bold font with an asterisk.

## Results

**Fig. 2:**
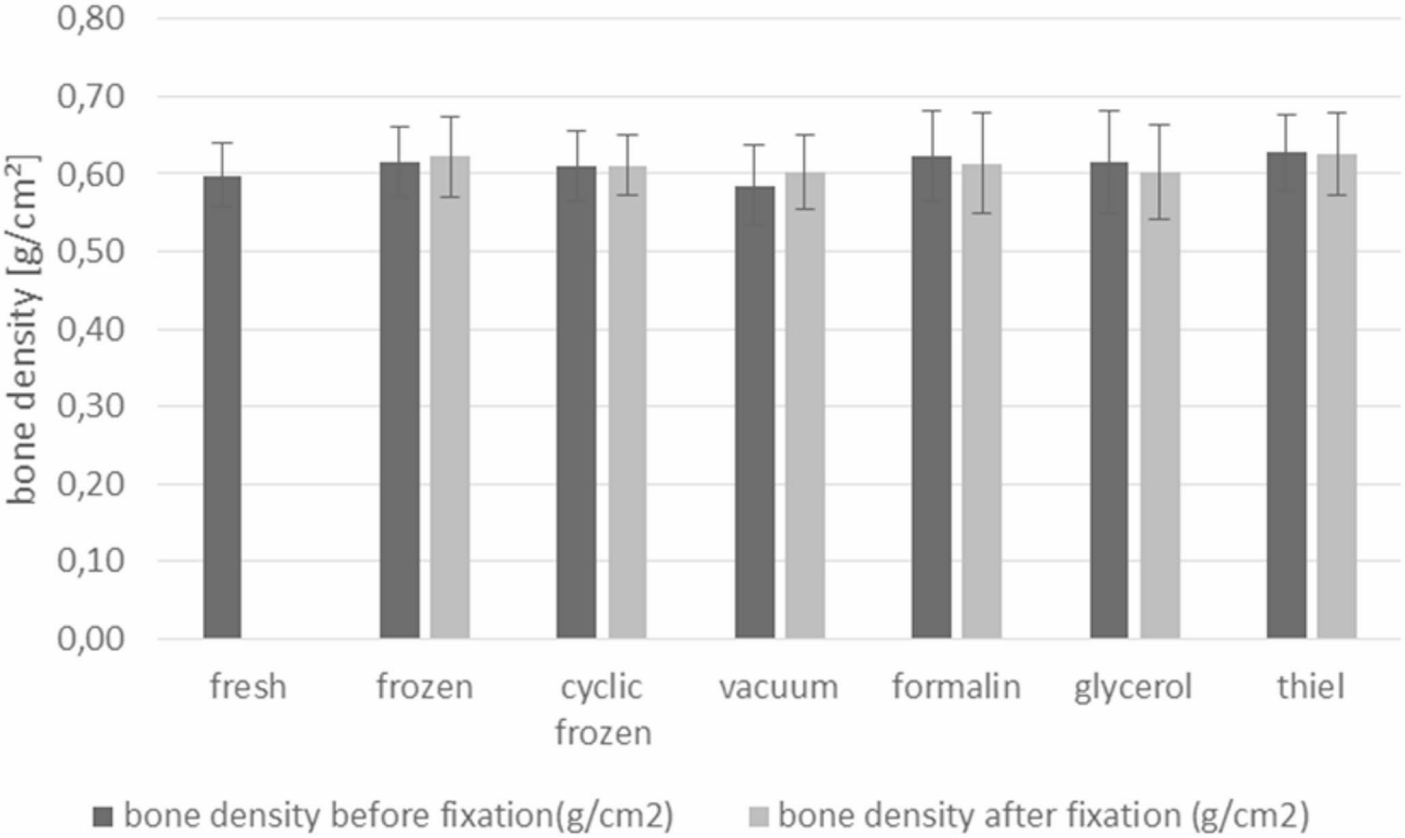
Bone density in dependence of conservations method. Metatarsals were analyzed before and after treatment using dual-energy X-ray absorptiometry (DXA)

Focussing on the influence of conservation method on bone density, our data show no evidence for loss of calcium during fixation procedure. As shown in Fig. 1, bone mineral density varied between 0.597 and 0.627 g/cm² before treatment, without significant differences between the groups. After conservation the value was between 0.602 and 0.626 g/cm² again without significant differences between the groups (Fig. 2).

In the three-point bending test, metatarsal bones displayed a consistent fracture pattern similar to AO classification A2.2. However, the number of attempts required to achieve 12 usable fractures varied depending on the conservation method. For physically preserved bones, the failure rate was between 25 and 50%, whereas chemically fixed bones had a failure rate below 15%, indicating a higher reproducibility with chemical preservation.

Regarding mechanical stability, preservation methods did not significantly affect either maximum load or breaking strength. However, deformation was significantly lower in bones treated with formalin or glycerol, resulting in a significant increase of stiffness (Table 1).

**Table 1:** Mechanical properties of bones after different preservation

	Maximum load (*N*)	deformation at maximum load (mm)	Stiffness at maximum load (*N*/mm)	Breaking strength (*N*)	deformation at break load (mm)	elastic–plastic transition point
Fresh	1083 ± 130	8.06 ± 1.70	137 ± 21	1012 ± 152	10.92 ± 4.07	678 ± 116
Fresh frozen	1142 ± 160	7.44 ± 1.18	156 ± 31	1041 ± 213	9.54 ± 3.76	857 ± 114
Multiple freeze/thaw cycles	1050 ± 128	7.57 ± 1.66	143 ± 28	987 ± 156	8.58 ± 2.74	684 ± 107
Vacuum at 4 °C	1020 ± 122	8.37 ± 1.23	123 ± 22	960 ± 162	10.18 ± 3.72	676 ± 169
Formalin fixation	1182 ± 220	5.69 ± 0.79* *p* = 0.000	210 ± 45* *p* = 0.000	1160 ± 217	5.76 ± 0.90* *p* = 0.000	652 ± 172
Glycerol fixation	1252 ± 263	6.55 ± 0.83	196 ± 64* *p* = 0.005	1215 ± 272	6.91 ± 2.27* *p* = 0.054	708 ± 164
Thiel Fixation	1104 ± 162	8.33 ± 1.40	137 ± 37	1016 ± 177	9.89 ± 2.85	726 ± 179

Displayed p-values refer to comparison between each preservation and fresh bone as control group. Bold and *-labeled values indicate statistically significant differences

In terms of osteosynthesis treatment, there were no difficulties performing the procedure across all groups. All fractures were easily reduced and fixed using a 5-hole-plate and screws.

After mechanical testing, an overall chi-square test suggested differences in the distribution of osteosynthesis failure patterns among the various preservation methods (Table 2). The observation that distal screw failures (screw positions 3 and 4) were more frequent in chemically preserved than in physically preserved specimens is exploratory and should be interpreted descriptively rather than as a direct group comparison.

**Table 2:** Failure pattern depending on conservation method

	Fresh	Fresh frozen	Cyclic frozen/ thaw	Vacuum at 4 °C	Formalin fixation	Glycerol fixation	Thiel fixation	*p*-value
Screw 1	7	7	7	8	6	8	4	0.699
Screw 2	5	5	5	10	3	5	6	0.147
Screw 3	0	2	4	0	7	6	4	0.003*
Screw 4	1	2	5	1	5	6	6	0.047*

Number represents torn screws at each position from proximal to distal. Bold and *-labeled values indicate statistically significant differences

Regarding the mechanical stability after osteosynthesis, force-based parameters are stable, but deformation-based parameters are not (Table 3).

**Table 3:** Mechanical properties of an osteosynthesis after different preservations

	Maximum load (*N*)	Deformation at maximum load (mm)	Stiffness at maximum load (*N*/mm)	Breaking strength (*N*)	Deformation at break load (mm)	Elastic–plastic transition point
Fresh	514 ± 117	9.34 ± 3.26	62 ± 30	479 ± 96	11.46 ± 3.70	295 ± 115
Fresh frozen	536 ± 121	8.47 ± 1.89	65 ± 15	476 ± 89	10.37 ± 2.63	325 ± 90
Multiple freeze/thaw cycles	620 ± 104	8.83 ± 1.72	72 ± 20	595 ± 98	9.51 ± 1.76	271 ± 138
Vacuum at 4 °C	499 ± 155	8.72 ± 2.48	60 ± 22	461 ± 125.	9.59 ± 2.37	298 ± 108
Formalin fixation	414 ± 160	5.97 ± 1.65* *p* = 0.003	72 ± 31	378 ± 164	**5.58** ± 1.42* *p* = 0.000	187 ± 62* *p* = 0.015
Glycerol fixation	708 ± 227	7.69 ± 1.29	96 ± 47* *p* = 0.013	679 ± 231* *p* = 0.033	8.35 ± 1.43* *p* = 0.023	316 ± 144
Thiel Fixation	521 ± 207	7.73 ± 1.71	70 ± 34	504 ± 201	8.40 ± 1.59* *p* = 0.027	290 ± 176

Displayed p-values refer to comparison between each preservation and fresh bone as control group. Bold and *-labeled values indicate statistically significant differences

## Discussion

In the context of our investigation, we first considered whether the different preservation methods might influence bone quality as reflected by bone mineral density. Based on our DXA measurements, which showed no statistically significant differences in bone density between groups, an adequately balanced randomization across the preservation methods can be assumed.

Bone density measurements showed no effect of the preservation or fixation method, in agreement with van Haaren et al. [21], Filipov et al. [22] and Kühlein et al. [23]. In contrast to Tornetta et al. [24], no impact of altered bone density on osteosynthetic fixation was detected, suggesting that the chemical fixation methods used do not measurably change bone mineralization [25, 26], at least within the investigated time frames.

Analysis of different physical conservation techniques revealed no significant difference between fresh bones and single freezing or several freeze/thaw cycles. This finding is in accordance with Linde et al. and Panjabi et al. [2, 27] who also reported comparable mechanical properties after freezing bones for various durations. A decrease in stiffness, as described by Sonstegard and Matthews [3], was not observed in our data. Instead, we found a slight, though statistically insignificant, increase in stiffness similar to observations by Pelker et al. [4]. This suggests that ice crystal formation, as previously proposed [3], plays only a minor role in the mechanical degradation of frozen bones—provided that freezer burn is prevented. Thus, physical preservation methods do not appear to have a relevant influence on mechanical stability.

In contrast, chemically preserved bones exhibited distinct mechanical alterations.

Bone mineral density—an important factor influencing the stability of osteosynthesis [24]—remained unaffected by chemical fixation in our study. This is consistent with van Haaren et al. [21], who also reported preserved mechanical properties after long-term storage, but contrasts with Filipov and Gueorguiev [22], who observed a reduction of mechanical stability. The increased stiffness in our chemically fixed specimens is more likely attributable to formaldehyde-induced protein cross-linking within the collagen matrix. This interpretation is supported by Burkhart et al. [12] and Goh et al. [28], who both described increased stiffness in diaphyseal bone following formalin fixation. The formation of methanoic acid may lead to partial mineral leaching [25], but this effect appears to be minor and time dependent [29]. More plausibly, the reduction in elasticity results from formaldehyde-mediated cross-links between peptide amino groups [26, 30], which limit the natural deformation capacity of the collagen network.

Interestingly, our results indicate that chemical fixation does not impact maximum load or breaking strength of the bone. It is important to note that this does not necessarily mean that the groups are identical in their characteristics. The absence of significant differences can have numerous causes, for example a small effect, that could not be detected.

According to our results in terms of these parameters fresh frozen and formalin-fixed bones appear to be equally suitable for mechanical testing, in line with findings by Topp et al. [14] and Zech et al. [31].

However, significant changes were observed in stiffness and deformation behavior, which may be attributed to the different loading protocols used in other studies (axial deformation vs. bending tests). These alterations confirm that chemically preserved bones may yield valid force-based results but are less reliable for deformation-based parameters.

Comparable findings have been reported for tendons and soft tissues. Hohmann et al. [30] compared conservation techniques for biceps tendons and found similar failure loads for Thiel- and formalin-preserved tissue as well as fresh-frozen tissue, yet an increased stiffness in chemically preserved samples. Thiel fixation, in particular, can alter collagen conformation through boric acid [32], potentially softening tissue as observed by Liao et al. [33] and Wilke et al. [34]. In our study, Thiel-fixed metatarsals showed only minor mechanical differences compared with physically preserved bones, suggesting that Thiel fixation may serve as a viable compromise between tissue preservation and mechanical fidelity. However, after osteosynthesis, constructs in Thiel-fixed bones failed earlier than in fresh specimens, indicating limitations in their load-bearing performance.

Regarding the pattern of failure, the reduced plastic deformation and altered energy absorption capacity due to matrix changes [25, 26], together with the lower dissipation of load peaks during bending of the construct—which now concentrate at the plate–screw interface, particularly at the distal screws where bending moments are highest, instead of resulting in global deformation—may explain the observed pattern of failure in our study.

Overall, our findings demonstrate that neither freezing nor vacuum storage alters the mechanical stability of bones or osteosynthesis constructs. Chemically fixed bones, however, exhibited increased stiffness and more reproducible fracture patterns, which can be advantageous for standardized mechanical testing. Nevertheless, formalin and glycerol fixation should be used cautiously, as they substantially alter deformation behavior. Thiel fixation appears to offer a partial exception, balancing preservation quality with near-physiological mechanical properties.

In summary mechanical testing focused on load-bearing capacity, both fresh-frozen and chemically fixed specimens are acceptable. However, deformation-dependent parameters should be interpreted with caution when using chemically fixed bones.

### Limitations

Porcine metatarsals differ from human ones in thickness and age (juvenile vs. elderly donors with bone loss), limiting direct interspecies comparability despite the study’s focus on preservation methods.

A potential limitation arises from the use of both left and right porcine metatarsals, which may have resulted in inconsistent lateral orientation during DXA measurements despite standardized positioning of the plantar surface and distal-to-proximal scanning direction.

## Data Availability

No datasets were generated or analysed during the current study.
